# Is ongoing testosterone required after pubertal induction in Duchenne muscular dystrophy?

**DOI:** 10.1530/EC-23-0245

**Published:** 2023-10-26

**Authors:** Claire L Wood, Kieren G Hollingsworth, Edrina Bokaie, Eric Hughes, Robert Muni-Lofra, Anna Mayhew, Rod T Mitchell, Michela Guglieri, Joseph McElvaney, Timothy D Cheetham, Volker Straub

**Affiliations:** 1 Department of Paediatric Endocrinology, Royal Victoria Infirmary, Newcastle upon Tyne, UK; 2 Translational and Clinical Research Institute, Faculty of Medical Sciences, Newcastle University, Newcastle upon Tyne, UK; 3 John Walton Muscular Dystrophy Research Centre, Newcastle University and Newcastle Hospitals NHS Foundation Trust, Newcastle upon Tyne, UK; 4 MRC Centre for Reproductive Health, The University of Edinburgh, Queens Medical Research Institute, Edinburgh, UK

**Keywords:** puberty, Duchenne muscular dystrophy, testosterone, muscle MRI

## Abstract

Glucocorticoids (GCs) reduce inflammation and preserve muscle function in boys with Duchenne muscular dystrophy (DMD) but cause pubertal delay. Pubertal induction with testosterone is recommended but longer-term outcome is unknown.

**Objective:**

To assess hypothalamic–pituitary–gonadal axis, muscle volume and function 5 years after pubertal induction.

**Methods:**

A prospective observational follow-up of a clinical study was conducted. 15 GC-treated males with DMD were treated with incremental testosterone for 2 years (end of regimen +2 years) then evaluated at +2.5 years and +5 years (final follow-up ~3 years after last injection). Data collected included testicular volume (TV), gonadotrophin, testosterone, inhibin B, muscle function, and limb muscle MRI.

**Results:**

Participants were 18.7 years (s.d. 1.6) at the final follow-up and had been on GC for 11.2 years (s.d. 2.2). Testosterone levels were similar at +2.5 years (8.6 nmol/L (s.d. 3.4) and 5 years (11.0 nmol/L (s.d. 6.1). TV increased from 2.8 mL (s.d. 0.9) at +2 years to 7.1 mL (s.d. 1.8) then 10.6 mL (s.d. 3.5) at +2.5 years and +5.0 years (*P* < 0.001). Inhibin B levels increased from 55.6 pg/mL (s.d. 47.0) at baseline to 158.2 pg/mL (s.d.87.6), *P* =0.004 at 5 years but remained lower than reference values (mean 305 pg/mL). Muscle contractile bulk decreased.

**Interpretation:**

Pubertal induction with testosterone in DMD is associated with HPG axis activation and ongoing increases in inhibin B, TV, and testosterone concentrations. Some patients have normal levels which is promising regarding future fertility. Given the beneficial impact of testosterone on bone health, muscle, and well-being, monitoring testosterone levels in this population and supplementation of sub-optimal levels is important.

## Introduction

Duchenne muscular dystrophy (DMD) is characterised by the absence of dystrophin protein and results in muscle cell fragility, inflammatory change, and the accumulation of fibrotic tissue and fat in skeletal muscles (1). Despite intensive research there is no cure for DMD and long-term high dose glucocorticoids (GCs) form the mainstay of treatment. GCs stabilise muscle strength for a period of time and have beneficial effects on the heart, lungs and spine (2). Chronic GC use, however, can result in many adverse effects including pubertal delay. Recent work at our centre has shown that incremental monthly testosterone injections can induce puberty in adolescents with DMD and delayed puberty (3). The 2-year testosterone regimen had a positive impact on the skeleton and contractile muscle bulk. There was also evidence of a beneficial impact on the underlying disease process, with reduced muscle inflammation as detected by a reduced T2 relaxation time on MR spectroscopy. Consideration for testosterone treatment for the induction of puberty has recently been introduced as part of the International Care Considerations in DMD (4). It remains unclear what happens to endogenous testosterone levels once testosterone supplementation is stopped, particularly in the context of ongoing GC therapy. Having shown a beneficial effect of testosterone on muscle, bone, and quality of life, it is important to explore whether these young men benefit from ongoing testosterone supplementation. There are, in addition, no data regarding spermatogenesis and fertility in the adult DMD population, other than anecdotal reports of adults fathering children. This study investigates the longer-term outcome and androgen status after pubertal induction with testosterone administered as part of a clinical trial (NCT02571205).

## Patients and methods

Participants were invited to take part in this observational follow-up study if they had been enrolled in the initial single-centre study that followed 15 prepubertal males with DMD who were aged between 12 and 17 years old. All were treated with a 2-year incremental regimen of 4-weekly testosterone injections (NCT02571205). Full details of the clinical trial have been published (3, 5). In this current study, the primary objective was to assess key components of hypothalamo–pituitary–gonadal (HPG) function following completion of the course of testosterone injections. The outcome measures in this observational follow-up study were therefore clinical and biochemical markers of HPG activity including inhibin B concentrations. The initial intention was to follow the participants on two further occasions, at 1 and 2 years after that visit. However, as a result of the coronavirus disease 2019 (COVID-19) pandemic and patients with DMD being required to shield, these visits could not be completed as planned. They were evaluated at 6 months after last injection as part of their clinical care and subsequently attended a final follow-up visit at a mean of 2.8 years (s.d. 0.40) after final injection. For the purposes of clarity, these timepoints are as follows:

- Baseline (0 years, at initiation of testosterone regimen)
- End of regimen (after 2 years of testosterone treatment or timepoint +2.0 years)
- 6 months after testosterone treatment completed (2.5 years from baseline or timepoint +2.5 years)
- Final follow-up (3 years after finishing testosterone treatment and 5 years from baseline or timepoint +5.0 years)

The outcome measures were as follows:

- Auxological assessment including height, weight, Tanner staging of pubertal status, testicular volumes (reported as mean values)
- Biochemical assessment of androgen status
- Bone age x-ray (using left wrist)
- Muscle cross-sectional area (CSA), contractile cross-sectional area (cCSA), fat fraction (FF), and T2 relaxation time (upper limb only) determined by muscle MRI of upper and lower limbs, motor performance evaluated using North Star Ambulatory Assessment (NSAA) and / or Performance of Upper Limb (PUL 2.0)
- Bone mineral adjusted density of the lumbar spine and total body (minus head) and body composition using dual-energy x-ray absorptiometry (DXA)

### Clinical assessment

Height and weight were measured using a stadiometer. Subjects who were unable to stand with their heels flat or who lost standing ability during the course of the follow-up had arm span recorded instead and used as a surrogate for height. For further information, please refer to the published protocol (5). Testicular size was assessed using a Prader orchidometer and pubertal staging determined using Tanner staging criteria (6). The bilateral testicular volume was calculated using the sum of the left + right volumes.

## Biochemical gonadal/androgen status

Biochemical pubertal status was determined by measuring markers of the following:

1. Leydig cell function – morning (pre-10:00 h) serum testosterone
2. Sertoli cell function – anti-Mullerian hormone (AMH) and inhibin B levels, which were analysed by ELISA after transportation to the Protein Reference Unit in Sheffield
3. Gonadotrophin function – luteinising hormone (LH) and follicle-stimulating hormone (FSH) concentrations as previously described (5)

### Bone age

Bone age was assessed using a radiograph of the left wrist that was then reported by a radiologist using the Greulich and Pyle method (7).

### Motor function and muscle strength

All participants performed PUL (Version 2.0) for assessing upper limb performance (8) and ambulatory patients also undertook the North Star Ambulatory Assessment for DMD (NSAA) (9).

### Bone mineral density

DXA scans were conducted using a Lunar iDXA (GE Lunar Corp, Madison, WI, USA) to assess lean vs total body mass (lean and fat mass combined) and enable calculation of bone mineral content (BMC), adjusted for age, size, and gender.

The patient’s height measured in the clinic visit (as detailed above) was used for size adjustment. Predicted lean body mass (LBM) for height was adjusted for sex and estimated Tanner stage. Stepwise linear regression was used with published reference ranges to obtain age, size and sex adjusted adjusted *Z*-scores for total body (minus head) BMC and Lumbar spine (L2-4) BMC for LBM (10).

### MRI data acquisition and image processing

Imaging was performed on a 3.0 Tesla scanner (for detailed methods, see section on supplementary data given at the end of this article). Briefly, quantitative Dixon FF imaging was collected using fivefold compressed sensing acceleration (11, 12). For the upper limb, quantitative Dixon FF imaging and T2 relaxation time imaging (a surrogate of muscle inflammation (13)) were performed and maps processed, modelling for the presence of fat. Regions of interest (ROIs) were defined for the upper and lower limb for multiple image slices and FF and T2 relaxation times were calculated together as area-weighted averages. CSA and cCSA were also calculated to quantify the area of viable muscle tissue remaining (14) (see supplementary material). A total FF, CSA, and cCSA was also calculated for the leg and arm of each participant by a weighted average of all the individual muscle groups in that limb. Results are presented by muscle group across all participants. Six healthy male controls, matched by bone age at baseline (as all participants in the trial had delayed puberty and bone age), underwent the same MRI protocol at baseline in the original study; these data were used as an initial comparator in this follow-up study.

### Statistical analysis and approvals

This was an observational study and the recruitment target reflected the number of boys who were enrolled in the original clinical trial. All data are presented as mean ± s.d. unless stated otherwise. Baseline and ‘end of regimen’ data from the original testosterone study has been used in the longitudinal analysis (3). Paired* t*-tests (or Wilcoxon rank-sum techniques if variables not normally distributed) were used to compare outcome measures during longitudinal analysis. Statistical significance was taken to be *P* < 0.05. Stata v15 was used for statistical analysis (Stata Statistical Software, release 14; StataCorp).

The study was approved by the Newcastle upon Tyne Research Ethics Committee. Informed, written consent was obtained from the participants.

## Results

### Baseline characteristics

Fifteen participants completed the initial testosterone clinical study (3). The last participant’s final visit of the original study was February 2019. All participants from the original study consented to take part in the follow-up study. They had been on GC therapy for a mean of 13.1 years (s.d.) by the final visit (+5.0 years). The GC regimen remained unchanged during both the regimen and follow-up for all participants. Nine participants were on deflazacort (mean GC dose 0.55 mg/kg; s.d. 0.13) and 6 on prednisolone (mean 0.47 mg/kg; s.d. 0.13) (Table 1). One subject was diagnosed with severe anxiety during the COVID-19 pandemic and as a result felt unable to attend for the full study visit. He had pubertal assessment and bloods taken, the results of which have been included in the analysis. Three participants required aeroplane travel to attend the study centre and so could not have a full assessment performed due to COVID-19 restrictions. Remote consultations were carried out for these participants, including pubertal assessment performed by the local doctor, but this did not include bone age x-ray, MRI, or functional measures.

**Table 1:** Individual patient characteristics.

	Baseline age (years)	Baseline bone age (years)	Final age (years)	Final bone age (years)	GC regimen	GC daily dose (mg/kg)	Initial NSAA	Final NSAA	Initial PUL	Final PUL
	**T+0y**	**T+0y**	**T+5y**	**T+5y**			**T+0y**	**T+5y**	**T+0y**	**T+5y**
1	14.8	8	18.7	15	D daily	0.52	7	2	36	21
2	13.3	8	17.4	11	P daily	0.67	4	NA	26	17
4	14.8	11	20.1	15.5	P daily	0.59	11	NA	41	22
5	12.4	12.5	17.6	16	D daily	0.44	NA	NA	34	35
6	14.5	8	19.6	15.5	P daily	0.33	NA	NA	38	20
7	16.3	11	21.4	15	P daily	0.48	NA	NA	26	18
8	12.1	6	17.2		D daily	0.5	16	ND	40	ND
9	12.3	10	17.2		D daily	0.49	30	ND	42	42
10	16.8	12	21.9		D daily	0.69	4	NA	20	ND
11	12.8	9.5	17.7		P 10 on/off	0.38	23	ND	41	39
12	14.2	6	18.8	13.5	D daily	0.69	13	ND	28	19
13	14.8	10	19.5	17.5	P daily	0.38	23	16	40	38
14	12.3	12.5	16.8	16	D daily	0.3	1	NA	31	16
15	13.1	9	17.6	15	D daily	0.62	26	ND	31	24
16	13.1	7	17.7	–	D daily	0.69	6	5	35	ND

D, deflazacort; P, prednisolone; NA, non-ambulant; ND, not done; NSAA, North Star ambulatory assessment; PUL, performance of the upper limb; T, testosterone.

Baseline (T+0y) denotes time 0 years at start of 2-year testosterone regimen. Final (T+5y) denotes time 5 years at end of extended follow-up, approx. 3 years after cessation of testosterone regimen.

### Pubertal status

All subjects were Tanner stage G5, P5 at the final follow-up (timepoint +5.0 years). Bilateral testicular volume significantly increased from 5.6 mL (s.d. 0.9) at the end of pubertal induction with testosterone (timepoint +2.0 years) to 13.8 mL (s.d. 1.8) at 6 months afterwards (timepoint +2.5 years) to 21.2 mL (s.d. 3.5) at the final follow-up (timepoint +5.0 years) (*P* < 0.001 for difference between end of regimen, +2.0 years, and final follow-up, +5.0 years). At the end of regimen (timepoint +2.0 years), the mean testosterone level was 10.5 nmol (s.d. 7.5). Testosterone levels remained similar at 6 months after the last injection (timepoint +2.5 years; 8.6 nmol/L s.d. 3.4) and final follow-up at 5.0 years (10.3 nmol/L s.d. 6.5, *P* = 0.09), but with a wide variability from <1–26.7 nmol.

LH/FSH levels were suppressed at the end of the 2-year testosterone regimen (median LH < 0.1 IU/L (IQR 0.1–0.1), median FSH < 0.1 IU/L (IQR 0.1–1.2)), but activation of the hypothalamo-pituitary axis was evident during follow-up, with LH and FSH levels increasing to 5.3 IU/L (IQR 3.2–5.9) and 7.9 IU/L (IQR 5.1–10.4) respectively by 6 months after the last injection (timepoint +2.5 years). Gonadotrophins remained similar at the final follow-up; LH was 3.9 IU/L (IQR 1.7–6.9) and FSH 4.7 IU/L (IQR 2.2–7.0, *P*-value for difference between end of regimen, timepoint +2.0 years and final follow-up, timepoint +5.0 years was 0.03 and 0.02, respectively).

A significant increase in inhibin B levels occurred from baseline to final follow-up (55.6 pg/mL (s.d. 47.0) to 158.2 pg/mL (s.d. 87.6), *P* = 0.004) but final levels were lower than the age- and sex-matched reference values (mean 305 pg/mL; IQR 240–445 pg/mL) (15).

AMH levels decreased significantly from a median of 437 pmol/L (IQR 185–640) at baseline to 77.7 (31.8–934) at the final follow-up (*P*-value for difference = 0.02), a value that is similar to the median value for 18-year-old men reported in a population-based study (median 55.6 pmol/L) (16).

One participant (highlighted with the letter ‘a’ in Table 2) had restarted testosterone supplementation during the final year of follow-up because his testicular volume had not increased, and his testosterone level remained low. He had been given one injection of 1 g testosterone undecanoate (Nebido) by the time of his follow-up study visit and was due an injection; therefore, his data were included in the analysis.

**Table 2:** Pubertal characteristics and gonadal function.

	T at end of regimen (nmol/L)	T 6 months after end regimen (mL)	T at final F/U (nmol/L)	LH at end of regimen (IU/L)	LH 6m after end regimen (IU/L)	LH at final F/U (IU/L)	FSH at end of regimen (IU/L)	FSH 6 m after end regimen (IU/L)	FSH at final F/U (IU/L)	Testicular vol at end of regimen (mL)	Testicular vol 6 months after end regimen (mL)	Testicular vol at final F/U, L,R (mL)
	**T+2y**	**T+2.5y**	**T+5y**	**T+2y**	**T+2.5y**	**T+5y**	**T+2y**	**T+2.5y**	**T+5y**	**T+2y**	**T+2.5y**	**T+5y**
1	10	14.7	17.5	0.1	3.2	5.9	1.4	5.5	4.7	3 (1, 2)	9 (4.5, 5)	24 (12, 12)
2	18.7	11.1	15.6	0.1	3.6	1.6	0.1	2.5	2.2	5 (2, 3)	20 (10, 10)	30 (15, 15)
4	10.3	9.7	26.7	4.9	3.2	8.8	13.1	11.1	6.8	6 (3, 3)	16 (8, 8)	16 (8, 8)
5^a^	8.3	5.7	8.0	0.1	2.6	0.6	0.1	4	1.5	6 (3, 3)	16 (8, 8)	12 (6, 6)
6^b^	17.9	11.7	6.2	0.1	13.7	3.0	0.1	32.9	18.1	8 (4, 4)	10 (5, 5)	20 (10, 10)
7	5.2	5.1	6.8	0.5	8.4	5.4	0.1	12.6	6.7	6 (3, 3)	16 (8, 8)	18 (8, 10)
8^b^	20.7	9.7	<1	0.1	5.9	7.1	0.1	6.5	1.6	6 (3, 3)	8 (4, 4)	18 (6, 6)
9	5.7	7.9	8.9	0.1	5.3	4.8	1.2	8.6	4.7	4 (2, 2)	20 (10, 10)	30 (15, 15)
10^b^	6.9	3.8	6.9	0.1	13.5	11.7	0.1	9.1	17.7	5 (2, 3)	8 (4,4)	12 (6, 6)
11	6.8	10.1	12.1	0.1	5.5	6.9	0.1	8.4	6.8	6 (3, 3)	–	30 (15, 15)
12	8.9	9.8	10.3	0.1	4.2	0.9	0.1	5.1	2.3	10 (5, 5)	10 (5, 5)	20 (10, 10)
13^b^	7.4	8.3	6	0.1	3.1	1.7	0.7	2.0	2.2	4 (2, 2)	–	30 (15, 15)
14^b^	31.4	4.9	4.4	0.1	5.7	5.3	0.1	10.4	14.4	5.5 (2.5, 3)	–	12 (6, 6)
15	4	4.8	8.4	0.1	2.9	3.1	0.1	7.8	7.0	4 (2,2)	15 (7, 8)	20 (10, 10)
16	5.2	12.8	16.1	6.4	5.3	2.8	7.2	7.9	3.4	6 (3, 3)	12 (6, 6)	24 (12, 12)
Mean (s.d.)/median (IQR)	10.5 (7.5)	8.6 (3.4)	11.0 (6.1)	<0.1 (0.1, 0.1)	5.3 (3.2, 5.9)	3.9 (1.7, 6.9)	<0.1 (0.1, 1.2)	7.9 (5.1, 10.4)	4.7 (2.2, 7.0)	5.6 (0.9)	13.8 (1.8)	21.2 (3.5)

FSH, follicle stimulating hormone; LH, luteinising hormone; T, testosterone.

^a^started Nebido supplementation during follow-up; ^b^recommended to start testosterone supplementation following final study visit.

Following assessment as part of this study, a further five participants were commenced on long-term testosterone supplementation, based on a clinical and biochemical assessment that included suboptimal testosterone values (<8 nmol/L) and a decreasing testosterone level. These are highlighted in Table 2 with the letter ‘b’.

### Muscle function

At the time of the last testosterone injection, 7/15 participants were still ambulant, by final visit (3 years later) only 4/15 remained ambulant (Table 1). The PUL score did not significantly change from 28.6 (s.d. 8.0) at the end of regimen to 25.9 (9.7) at the final follow-up (*P* = 0.5), in contrast to natural history data in DMD which shows that PUL 2.0 is sensitive to detect the typical deterioration seen in the decline phase over a 2-year period (17).

### Anthropometric variables

Height *Z*-scores remained similar throughout, −3.2 (s.d. 1.3) at baseline, −3.4 (s.d. 1.1) at the end of regimen at timepoint +2.0 years and −3.2 (s.d.1.0) at the final follow-up. The mean height gain was 16.7 cm (s.d. 9.9) during the overall 5-year period, giving a mean final height of 151.4 cm (s.d. 9.1). BMI *Z*-score was 1.6 (s.d. 1.4) at the end of the testosterone regimen and significantly lower at 1.5 (s.d. 1.4) by final follow-up (*P*-value for difference between baseline and final follow-up = 0.04). Fat and lean mass indices remained similar during the course of the studies (Table 3).

**Table 3:** Anthropometric variables. Outcome variables at baseline, after 2-year incremental regimen of intramuscular testosterone and after 3 further years of follow-up.

	Baseline	End of regimen mean (s.d.)	Final F/U
	T+0y	T+2y	T+5y
Height (cm)	133.6 (10.7)	142.4 (7.4)	151.4 (9.1)
Height *Z*-score	−3.17 (1.3)	−3.44 (1.1)	−3.2 (1.0)
Target height (cm)	183.3 (4.6)		
Weight (kg)	48.3 (12.4)	52.8 (12.2)	59.6 (15.7)
Weight *Z*-score	−0.19 (1.6)	−0.94 (1.8)	−1.0 (1.8)
BMI (kg/m^2^)	26.8 (4.5)	26.3 (5.0)	26.5 (7.8)
BMI *Z*-score	2.16 (0.9)	1.64 (1.35)	1.5 (1.4)
Lean mass index (kg/m^2^)	12.4 (2.3)	12.8 (2.9)	11.5 (2.7)
Fat mass index (kg/m^2^)	13.6 (4.2)	12.5 (4.3)	14.1 (6.1)
NSAA (if ambulant at both timepoints)	18 (8.9)	13 (7.23)	8 (7.4)
PUL	33.9 (6.7)	30.9 (8.7)	25.9 (9.7)
LS BMAC *Z*-score	0.2 (2.2)	0.4 (2.2)	−2.3 (1.1)

### Bone mineral density

After 2 years of testosterone treatment (+2.0 years), LS BMC *Z*-score was 0.4 (s.d. 2.2, range −2.8 to +4.0). By final follow-up (+5.0 years) it was significantly lower at −2.3 (s.d. 1.0, range −3.5 to −0.4, *P* < 0.001). Six patients were on bisphosphonates at the beginning of the follow-up period (two were on 6 monthly zoledronic acid infusions, three on weekly risedronate, and one on weekly alendronate, who switched during the study from risedronate to effervescent as unable to swallow tablet. No patients stopped their bisphosphonates during the study follow-up.

### Bone age

Bone age advanced in all patients during the extension study period but remained delayed overall. The mean bone age at the final follow-up was 15.0 years (s.d.1.7) compared to a chronological age of 18.7 years (s.d. 1.6).

### Muscle MRI

#### Longitudinal analysis of fat fraction in upper and lower limbs

Fat fraction (FF) was higher in every muscle group, and for total FF, when comparing healthy controls and participants with DMD at all assessments (Table 4). A significant increase in total FF of the arms and individually in the upper arm extensor group (UEG) and upper arm flexor group (UFG) groups occurred between the end of regimen (+2.0 years) and final follow-up (+5.0 years) with the largest increase seen in the UFG (11.8%, *P*-value for difference between +2.0 years and +5.0 years ⋜ 0.01) (Table 4). UEG and UFG values were also significantly greater at 5 years compared to baseline (*P* < 0.05). FF also significantly increased in all calf and thigh muscle groups from end of regimen to final follow-up with the greatest increase seen in the biceps femoris long head (13.8% increase, *P* < 0.01). In the calf, the soleus had the biggest percentage change in FF (10.3% increase, *P* < 0.01).

**Table 4:** Upper limb MRI.

Muscle group	Number	DMD baseline	DMD end regimen	DMD final F/U	Control
		T+0y	T+2y	T+5y	
T2 (ms)					
FEG	9	31.2	29.6	30.4	28.3^c^
UEG	10	32.8	31.4	32.5^a^	29.5^c^
FFG	9	31.6	29.7	31.1^a^	28.5^c^
UFG	10	30.7	30.2	30.9	29.1^b^
FF (%)					
FEG	10	11.3	10.5	12.4	3.9^b^
UEG	10	18.3	19.9	28.5^c,d^	3.4^c^
FFG	9	12.9	12.1	14.7	3.7^b^
UFG	9	20.2	26.9	38.7^c,d^	3.6^c^
Total	10	13.6	17.0	23.5^b^	3.6^c^
CSA (mm^2^)					
FEG	10	399.6	513.3	417.0	
UEG	10	874.4	975.6	914.4	
FFG	10	883.1	925.1	952.2	
UFG	10	592.3	668.4	635.4	
Total	10	2581.2	3082.5	2919.1	
cCSA (mm^2^)					
FEG	10	357.8	462.7	363.9^b^	
UEG	10	749.4	819.6	678.8^c^	
FFG	10	797.3	826.0	820.7	
UFG	10	499.9	513.1	396.9^b,d^	
Total		2253.2	2621.7	2260.4^b^	

*P*-values with letters ‘a’, ‘b’, ‘c’, and ‘d’ in DMD 5 years are paired* t*-tests vs 2 years; *P-*values with letter ‘e’ in DMD 5 years are paired *t*-tests vs baseline; *P*-values in the Control column are unpaired* t*-tests vs DMD 5 years.

^a^*P* < 0.06; ^b^*P* < 0.05; ^c^*P* < 0.01; ^d^*P* < 0.05.

FEG, forearm extensor group; FFC, forearm flexor group; T, testosterone; Total, combined muscle groups; UEG, upper arm extensor group; UFG, upper arm flexor group.

When comparing the baseline and 5-year data, an increase in total FF occurred in the leg (31.9–43.3%, *P* < 0.05) and individually in 4 out of 5 thigh muscle groups (rectus femoris, vastus lateralis, biceps femoris long head and sartorius, Table 5) and 2 out of the 5 calf muscle groups (medial gastrocnemius, soleus). The FF evolution for individual subjects can be seen in Fig. 2A (for arm) and Fig. 2C (for leg).

**Figure 1:**
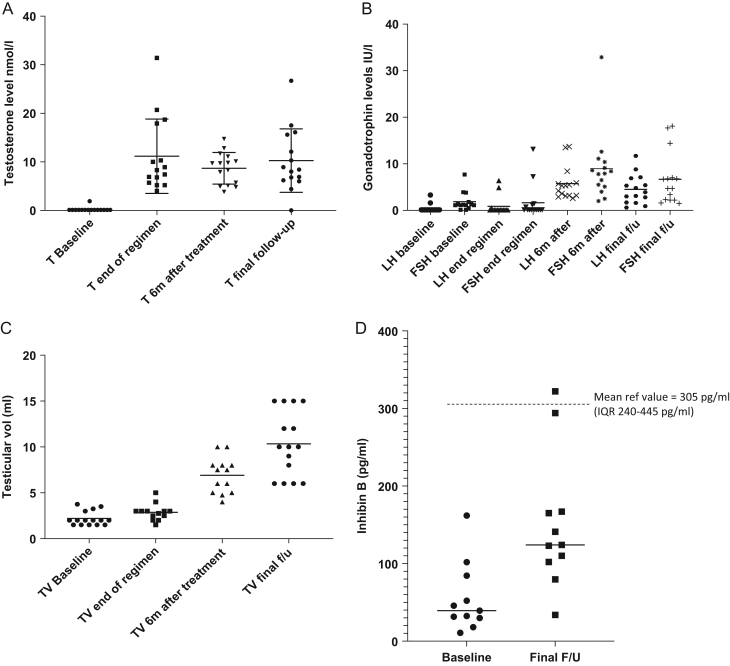
Gonadal function during pubertal induction and follow-up. (A) Testosterone (T) levels during initial study and extension. (B) Gonadotrophin levels during initial study and extension (LH, luteinising hormone; FSH, follicle-stimulating hormone). (C) Testicular volumes during initial study and extension (TV, testicular volume). (D) Inhibin B levels during initial study and extension.

**Figure 2:**
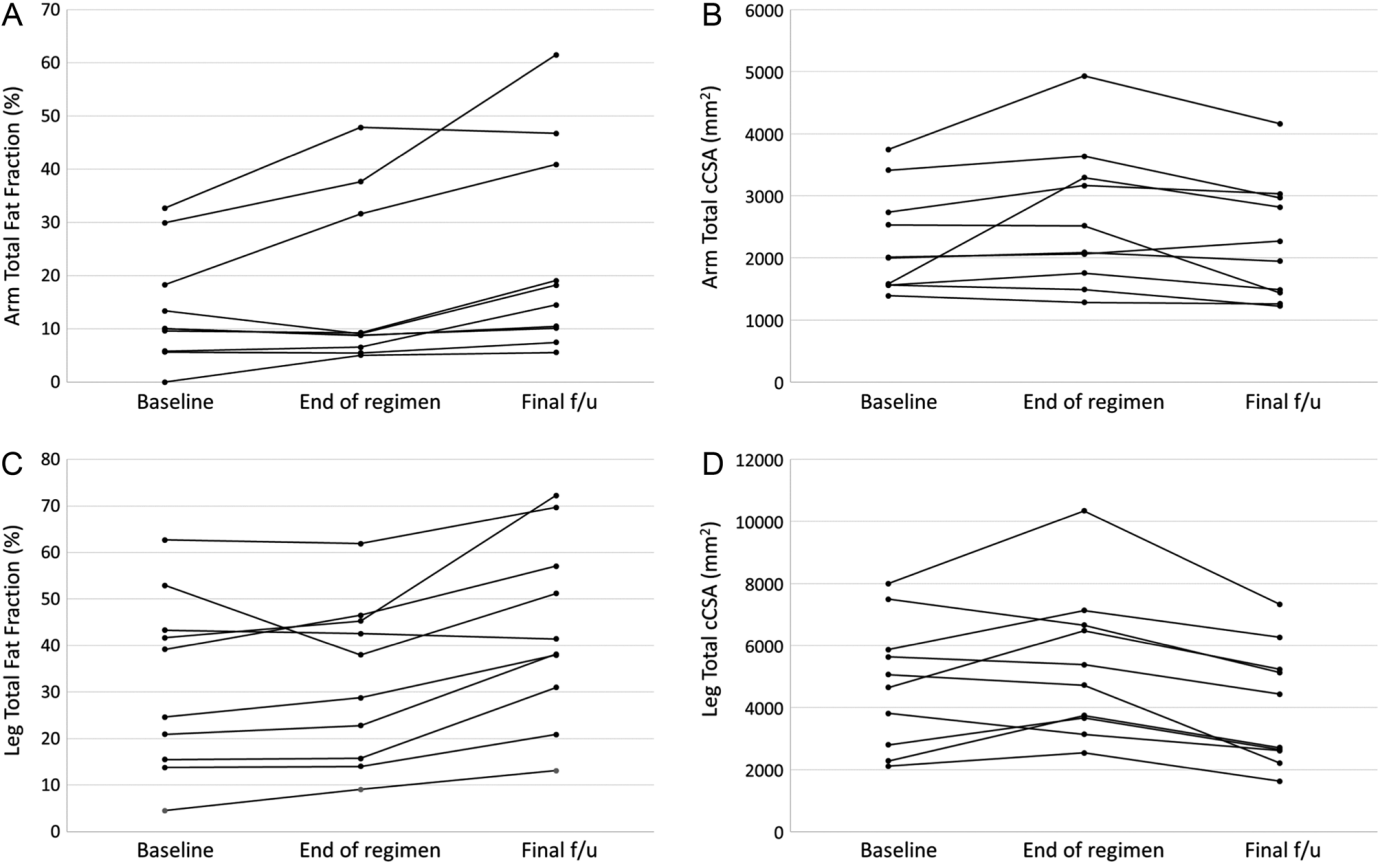
A) Arm total fat fraction and B) total cCSA measured by MRI shown for individual participants at baseline, end of regimen, and the final follow-up. C) Leg total fat fraction and D) total cCSA measured by MRI shown for individual participants at baseline, end of regimen, and the final follow-up.

**Table 5:** Lower limb MRI.

	Control	FF (%) *n* = 10			CSA (mm^2^) *n* = 10			cCSA (mm^2^) *n* = 10		
		BL	End regimen	Final F/U	BL	End regimen	Final F/U	BL	End regimen	Final F/U
		T+0y	T+2y	T+5y	T+0y	T+2y	T+5y	T+0y	T+2y	T+5y
TA	2.0^a^	11.6	11.0	17.4^a^	313.7	414.5	304.3	281.0	374.3	255.0^a^
MG	2.8^b^	22.2	20.3	29.7^a,d^	1297.7	1493.2	1143.4^b^	1024.5	1214.9	827.8^b,d^
LG	2.5^b^	25.2	24.0	34.1^a^	561.0	676.1	540.1	391.5	485.8	348.8^b,d^
SOL	2.4^b^	17.9	18.4	28.7^b,d^	1595.7	1662.9	1633.1	1306.9	1372.4	1170.6^a^
RF	1.6^c^	47.5	48.3	60.0^b,d^	568.6	647.3	654.6^a^	286.2	314.4	243.0^a^
VL	2.0^c^	48.1	51.4	60.4^a,d^	1069.0	1171.2	1056.8^b^	542.2	562.3	407.6^b,d^
BFLH	2.7^c^	54.9	58.0	71.8^b,d^	857.7	908.5	872.7^c^	355.5	390.1	259.3^c,d^
ST	2.7^b^	28.2	32.0	45.3^b^	554.7	678.0	601.8^b^	410.9	476.3	353.7^b^
SART	5.1^b^	27.8	31.6	44.2^a,d^	235.1	271.1	253.0^b^	173.3	191.5	157.2^b^
Total	2.4	31.9	32.5	43.3^a,d^	7052.1	7921.9	7059.2^a^	4771.9	5381.8	4023.2^c^

*P*-values with letters ‘a’, ‘b’, and ‘c’ in DMD 5 years (T+5y) are paired* t*-tests vs end of regimen (T+2y);

*P*-values with letter ‘d’ in DMD 5 years (T+5y) are paired *t*-tests vs baseline (T+0y);

*P*-values in the Control column are unpaired *t*-tests vs DMD 5 years.

^a^*P* < 0.05; ^b^*P* < 0.01; ^c^*P* < 0.001; ^d^*P* < 0.05.

BFLH, biceps femoris long head; LG, lateral gastrocnemius; MG, medial gastrocnemius; RF, rectus femoris (thigh); SART, sartorius; SOL, soleus; ST, semitendinosus; T, testosterone; TA, tibialis anterior (calf); Total, combined muscle groups; VL, vastus lateralis.

#### Longitudinal analysis of cross-sectional area in upper and lower limbs

Between the end of regimen and the final follow-up, there was no change in CSA for any of the arm muscle groups, or for the total CSA of the arm. CSA in the calf muscle groups remained stable, with a significant change between end of regimen and final follow-up only seen in medial gastrocnemius (349.8 mm² decrease, *P* < 0.01), whilst significant decreases in CSA were noted from end of regimen to final follow-up in all thigh muscle groups, except for RF where a small increase was noted (Table 5). The total leg CSA significantly decreased between end of regimen and final follow-up (*P* < 0.05).

#### Longitudinal analysis of contractile cross-sectional area in upper and lower limbs

There was a significant decrease in total cCSA of the arm between end of regimen and final follow-up (361.3 mm², *P* < 0.05) and also separately in the forearm extensor group (FEG), UEG and UFG muscle groups; with the largest decrease observed in UEG (140.8 mm², *P* < 0.01). There was a significant decrease in cCSA in UFG from baseline to final follow-up (*P* < 0.05). In the other arm muscle groups, the final follow-up cCSA values were similar to baseline.

A significant reduction in cCSA was also seen in all individual leg muscle groups of both the thigh and calf (Table 5) contributing to a decrease in total cCSA between end of regimen and final follow-up of 1358.6 mm^2^ or 25.2 % (*P* < 0.001, Table 5). Despite the significant increase that we observed between baseline and end of regimen in total cCSA for the leg (3), the final cCSA in lateral and medial gastrocnemius (calf) and vastus lateralis and biceps femoris long head (thigh) groups were significantly less than at baseline (*P* < 0.05 for difference between baseline and final follow-up). The evolution of cCSA for individual subjects can be seen in Fig. 2B (for arm) and Fig. 2D (for leg).

### T2 relaxation time

T2 relaxation time data was only collected for arm muscle groups due to time constraints and to ensure patient comfort within the MRI scanner. There was a trend for increase in T2 relaxation time for UEG and FFG from the end of regimen visit to final follow-up (*P* < 0.052 and *P* < 0.056, indicated in Table 4 as *P* < 0.06).

## Discussion

The newly revised standards of care highlight the importance of the timely recognition and management of pubertal delay in DMD (18). This study describes HPG activity in adolescents with DMD following the same 2-year pubertal induction regimen using incremental monthly injections of intramuscular testosterone. Rather than ongoing relative HPG axis inactivity, the testosterone given during the 2-year pubertal induction regimen appeared to promote gonadotrophin release in most participants, despite ongoing GC use. As such, GC does not appear to suppress gonadotrophin production to the extent that it does before puberty. There was ongoing HPG axis activity following testosterone therapy, but the clinical and biochemical parameters were relatively low when compared to age-related norms.

By the time of the final follow-up, when the mean age of participants was 18.6 years, one participant had already restarted testosterone supplementation and a further five were advised to restart after their final assessment (subjects marked ‘b’ in Table 2). They all had an increase in testosterone levels during follow-up but they had been deemed not to be sufficient (<8 nmol/L). Therefore, just under half of the study cohort required supplementation by 3 years after stopping their incremental regimen. The mean testosterone level at +5.0 years was 10.3 nmol/L, within the lower range of normal for a healthy adult male, but considerably lower than age-related normative mean levels of 15.4 nmol/L (18). There was also considerable variability with the values ranging from <1nmol/L to 26.7 nmol, which does not appear to be mutation specific or related to ambulation status, suggesting that there may be additional factors affecting the HPG axis including inter-individual sensitivity to GC. This highlights the need for ongoing surveillance after pubertal induction and for studies of larger cohorts to better understand the variability between individual responses.

The testicular volume of 10.6 mL at the final follow-up was significantly greater than at all other time points during the incremental testosterone regimen but remained considerably lower than mean reference bilateral values for the adult testis of 31 mL (19). Semen volume, sperm density, total sperm count, total motile sperm count, and serum FSH, LH, and testosterone levels have all been shown to correlate with testicular volume (20), although numbers in this study are too small to demonstrate such a correlation and no semen analysis was undertaken. Inhibin B is secreted from the Sertoli cells in the testis. Inhibin B levels significantly increased from baseline to 158.2 pg/mL (s.d. 87.6), but final levels remained lower than age- and sex-matched reference values of 305 pg/mL (IQR 240–445 pg/mL) (15). There was a significant positive correlation between testicular volume and inhibin B level at the final follow-up (T+5y), *r* ^2^ = 0.6. Inhibin B, FSH levels and testicular volumes have been shown to positively correlate with spermatogenesis and sperm density and the increase seen in both testicular volume and inhibin B during pubertal induction in this cohort of men with DMD is promising with respect to the prospect of future fertility, although semen analysis would be required to confirm sperm production. A proportion of participants also showed borderline elevation in FSH levels at the final follow-up (median 4.7 IU/L, IGR range 2.2–7.0) which may reflect feedback from the testis to pituitary gland and be consistent with sub-optimal spermatogenesis (21). Three of the 15 participants had FSH levels greater than 10.4 IU which have been shown to predict azoospermia in patients receiving gonadotoxic therapies (22).

As the results of this study and individual case reports (23, 24) suggest that spermatogenesis can be preserved in men with DMD following pubertal induction despite long-term, high-dose GC use, it would be important to consider whether a young man with DMD wishes to have children in the future before restarting long-term testosterone supplementation as this could have an impact on their fertility prospects. It may be that testosterone supplementation is appropriate until fertility is being considered and then the use of human chorionic gonadotrophin may be preferred; a technique which is utilised in the treatment of hypogonadotrophic hypogonadism (25).

The extended nature of this study has highlighted the inevitable decline in muscle function and reduction in lumbar spine bone density that is consistent with the late-ambulant/non-ambulant transition phase in DMD and has been shown in other studies (26) Given the known additional advantages of testosterone on bone health (27), muscle and well-being, it will be important to continue monitoring testosterone levels in this population and supplement as required if levels become sub-optimal. The British Society for Sexual Medicine guidelines suggest that a testosterone level <8 nmol/L should be treated, although it also depends on the clinical situation (28). Once treated it is recommended that a target testosterone level should be 15–30 nmol/L. Whilst this is guidance for the healthy population, there are clear advantages for men with DMD to have an optimal testosterone level, as our previous study shows (3).

Our previous publication identified significant increases in both CSA and cCSA of the total arm and leg muscle groups during the interventional study period (and some individual muscle groups), from baseline to end of regimen (+2 years), suggesting the development of new healthy muscle tissue, whilst FF remained stable in the arms and legs (3). In this current study, MRI data demonstrated a clear deterioration in muscle integrity after cessation of the testosterone treatment with increases in FF and either unchanged or reduced CSA leading to overall reductions in cCSA. Total cCSA, representing the residual functional muscle volume, increased during testosterone treatment (3) but decreased during the extended follow-up period after the end of the regimen. The MRI findings in this study highlight the importance of prompt intervention with testosterone to treat pubertal delay in DMD. The initial study findings described an unexpected increase in cCSA for arms and legs coupled with a reduction in T2 relaxation time for the arm, and no increases in total limb FF, which suggested that testosterone treatment improved muscle strength and perhaps also ameliorated the underlying inflammatory process in DMD. These findings were in contrast to several natural history MRI studies of DMD that show disease progression with time and an increase in FF with time in adolescents with DMD (14, 29, 30). It remains unclear whether exogenous testosterone confers an additional benefit in terms of contractile muscle bulk and inflammatory status of muscle in DMD or whether the 2-year incremental regimen was offered early enough to temporarily halt the later muscle decline. We propose to carry out further work in this area.

### Limitations of the study

Unfortunately, the COVID-19 pandemic had a considerable impact on data collection in this study and so the number of study visits was reduced from two to one. Despite this, all patients agreed to take part and data were collected consistently. The original cohort only contained 15 participants and therefore the size of the follow-up cohort was limited. The cohort is too small, in particular, to draw strong conclusions regarding the trajectory of the bone density data in relation to testosterone use. It is difficult to obtain accurate measures of height, as contractures and then loss of ambulation make standing height impossible, whilst ulnar length can overestimate height. This may have had an impact on the final DEXA *Z*-scores. In order to avoid this, both standing height and ulnar length/arm span were recorded in patients during the decline phase, so that consistent measurement techniques could be maintained.

## Conclusion

Earlier testosterone therapy is associated with ongoing HPG activity. The increase in inhibin B observed in this cohort of men with DMD is promising with respect to future fertility. Testosterone levels and testicular volumes, however, remain lower than adult reference values. Upper and lower limb muscle functional volume increased during the intervention but declined in the years after cessation of supplementation. Given the known additional advantages of testosterone on bone health, muscle, and well-being and the likelihood for a further reduction in HPG function associated with the inevitable health deterioration seen in young adults with DMD, a more proactive approach to ongoing testosterone supplementation may be appropriate following pubertal induction.

## Declaration of interest

Volker Straub is or has been on advisory boards for Astellas Gene Therapies, Biogen, Edgewise Therapeutics, Kate Therapeutics, ML Bio Solutions, Novartis Gene Therapies, Roche, Sanofi, Sarepta Therapeutics, Vertex Pharmaceuticals, and Wave Therapeutics. He received speaker honoraria from Pfizer, Sanofi, and Sarepta Therapeutics. He has research collaborations with Sarepta Therapeutics and Sanofi. Robert Muni Lofra has received speaker honoraria from Biogen and has been recently on advisory boards for Roche and Biogen. Anna Mayhew has served on medical/scientific advisory boards for Regenxbio, Sarepta, Biogen, and Roche and has received fees for consulting and training services from Biogen, Roche, Novartis, Biohaven, PTC, Sarepta, Italfarmaco, Dyne, Pfizer, Summit, Catabasis, Santhera, Vision, Lysogene, Modis, Amicus, Analysis Group, MDUK, and DUK. Michela Guglieri has received speaker honoraria from Sarepta, Novartis, and Italfarmaco and is or has been on advisory boards for PTC Therapeutics, Pfizer, NS Pharma, and Dyne. She has research collaborations with Sarepta and PTC Therapeutics. Kieren Hollingsworth performs commercial work with Astellas Gene Therapies. Claire Wood, Edeina Bokaie, Eric Hughes, Joseph Mcelvery, Rod Mitchell, and Tim Cheetham have no conflicts of interest.

## Funding

The study was generously funded by Duchenne UK. CW was funded by the Medical Research Council http://dx.doi.org/10.13039/501100000265/MDUK (MR/N020588/1). RTM is funded by a UK Research and Innovation http://dx.doi.org/10.13039/100014013 (UKRI) Future Leaders Fellowship MR/S017151/1.

## References

[1] Duan D Goemans N Takeda S Mercuri E & Aartsma-Rus A. Duchenne muscular dystrophy. Nature Reviews. Disease Primers 2021 7 13. (10.1038/s41572-021-00248-3) PMC10557455 33602943

[2] Matthews E Brassington R Kuntzer T Jichi F & Manzur AY. Corticosteroids for the treatment of Duchenne muscular dystrophy. Cochrane Database of Systematic Reviews 2016 5 CD003725. (10.1002/14651858.CD003725.pub4) PMC8580515 27149418

[3] Wood CL Hollingsworth KG Hughes E Punniyakodi S Muni-Lofra R Mayhew A Mitchell RT Guglieri M Cheetham TD & Straub V. Pubertal induction in adolescents with DMD is associated with high satisfaction, gonadotropin release and increased muscle contractile surface area. European Journal of Endocrinology 2021 184 67–79. (10.1530/EJE-20-0709) 33112266

[4] Birnkrant DJ Bushby K Bann CM Apkon SD Blackwell A Brumbaugh D Case LE Clemens PR Hadjiyannakis S Pandya S, ***et al***. Diagnosis and management of Duchenne muscular dystrophy, Part 1: diagnosis, and neuromuscular, rehabilitation, endocrine, and gastrointestinal and nutritional management. Lancet. Neurology 2018 17 251–267. (10.1016/S1474-4422(1830024-3) 29395989 PMC5869704

[5] Wood CL Cheetham TD Hollingsworth KG Guglieri M Ailins-Sahun Y Punniyakodi S Mayhew A & Straub V. Observational study of clinical outcomes for testosterone treatment of pubertal delay in Duchenne muscular dystrophy. BMC Pediatrics 2019 19 131. (10.1186/s12887-019-1503-x) 31023296 PMC6482579

[6] Marshall WA & Tanner JM. Variations in the pattern of pubertal changes in boys. Archives of Disease in Childhood 1970 45 13–23. (10.1136/adc.45.239.13) 5440182 PMC2020414

[7] Greulich WW & Pyle SI Radiographic Atlas of Skeletal Development of the Hand and Wrist, 2nd ed. Stanford: Stanford University Press 1959.

[8] Mayhew AG Coratti G Mazzone ES Klingels K James M Pane M Straub V Goemans N Mercuri E Ricotti V, ***et al***. Performance of Upper Limb module for Duchenne muscular dystrophy. Developmental Medicine and Child Neurology 2019 62 633–639. (10.1111/dmcn.14361) 31538331

[9] Mayhew AG Cano SJ Scott E Eagle M Bushby K Manzur A Muntoni F & North Star Clinical Network for Neuromuscular Disease. Detecting meaningful change using the North Star Ambulatory Assessment in Duchenne muscular dystrophy. Developmental Medicine and Child Neurology 2013 55 1046–1052. (10.1111/dmcn.12220) 23909763

[10] Crabtree NJ Kibirige MS Fordham JN Banks LM Muntoni F Chinn D Boivin CM & Shaw NJ. The relationship between lean body mass and bone mineral content in paediatric health and disease. Bone 2004 35 965–972. (10.1016/j.bone.2004.06.009) 15454104

[11] Hollingsworth KG Higgins DM McCallum M Ward L Coombs A & Straub V. Investigating the quantitative fidelity of prospectively undersampled chemical shift imaging in muscular dystrophy with compressed sensing and parallel imaging reconstruction. Magnetic Resonance in Medicine 2014 72 1610–1619. (10.1002/mrm.25072) 24347306

[12] Loughran T Higgins DM McCallum M Coombs A Straub V & Hollingsworth KG. Improving highly accelerated fat fraction measurements for clinical trials in muscular dystrophy: origin and quantitative effect of R2* changes. Radiology 2015 275 570–578. (10.1148/radiol.14141191) 25575118

[13] Carlier PG. Global T2 versus water T2 in NMR imaging of fatty infiltrated muscles: different methodology, different information and different implications. Neuromuscular Disorders 2014 24 390–392. (10.1016/j.nmd.2014.02.009) 24656605

[14] Ricotti V Evans MRB Sinclair CDJ Butler JW Ridout DA Hogrel JY Emira A Morrow JM Reilly MM Hanna MG, ***et al***. Upper limb evaluation in Duchenne muscular dystrophy: fat-water quantification by MRI, muscle force and function define endpoints for clinical trials. PLoS One 2016 11 e0162542. (10.1371/journal.pone.0162542) 27649492 PMC5029878

[15] Kelsey TW Miles A Mitchell RT Anderson RA Hamish WHB & Wallace B. A normative model of serum inhibin B in young males. PLoS One 2016 11 e0153843. (10.1371/journal.pone.0153843) 27077369 PMC4831823

[16] Jopling H Yates A Burgoyne N Hayden K Chaloner C & Tetlow L. Paediatric anti-Müllerian hormone measurement: male and female reference intervals established using the automated Beckman Coulter Access AMH assay. Endocrinology, Diabetes and Metabolism 2018 1 e00021. (10.1002/edm2.21) PMC6354749 30815559

[17] Mayhew AG Coratti G Mazzone ES Klingels K James M Pane M Straub V Goemans N Mercuri E & Pul Working Group. Performance of Upper Limb module for Duchenne muscular dystrophy. Developmental Medicine and Child Neurology 2020 62 633–639. (10.1111/dmcn.14361) 31538331

[18] Kelsey TW Li LQ Mitchell RT Whelan A Anderson RA & Wallace WHB. A validated age-related normative model for male total testosterone shows increasing variance but no decline after age 40 years. PLoS One 2014 9 e109346. (10.1371/journal.pone.0109346) 25295520 PMC4190174

[19] Rundle AT & Sylvester PE. Measurement of testicular volume. Its application to assessment of maturation, and its use in diagnosis of hypogonadism. Archives of Disease in Childhood 1962 37 514–517. (10.1136/adc.37.195.514) 13975542 PMC2012925

[20] Sakamoto H Ogawa Y & Yoshida H. Relationship between testicular volume and testicular function: comparison of the Prader orchidometric and ultrasonographic measurements in patients with infertility. Asian Journal of Andrology 2008 10 319–324. (10.1111/j.1745-7262.2008.00340.x) 18097521

[21] Gordetsky J Wijngaarden Van E & O’Brien J. Redefining abnormal follicle-stimulating hormone in the male infertility population. BJU International 2012 110 568–572. (10.1111/j.1464-410X.2011.10783.x) 22177092

[22] Kelsey TW McConville L Edgar AB Ungurianu AI Mitchell RT Anderson RA & Wallace WHB. Follicle stimulating hormone is an accurate predictor of azoospermia in childhood cancer survivors. PLoS One 2017 12 e0181377. (10.1371/journal.pone.0181377) 28727831 PMC5519149

[23] Action Duchene. Mitch Coles – “Living my life the way I want to live it”. London, UK: Action Duchenne, 2019. (available at: https://www.actionduchenne.org/stories/mitch-coles-living-my-life-the-way-i-want-to-live-it/)

[24] The Guardian. My lifelong desire. London, UK: The Guardian, 2007. (available at: https://www.theguardian.com/society/2007/jan/15/health.socialcare)

[25] Lee JA & Ramasamy R. Indications for the use of human chorionic gonadotropic hormone for the management of infertility in hypogonadal men. Translational Andrology and Urology 2018 7(Supplement 3) S348–S352. (10.21037/tau.2018.04.11) 30159241 PMC6087849

[26] Crabtree NJ Roper H & Shaw NJ. Cessation of ambulation results in a dramatic loss of trabecular bone density in boys with Duchenne muscular dystrophy (DMD). Bone 2022 154 116248. (10.1016/j.bone.2021.116248) 34718220

[27] Behre HM Kliesch S Leifke E Link TM & Nieschlag E. Long-term effect of testosterone therapy on bone mineral density in hypogonadal men. Journal of Clinical Endocrinology and Metabolism 1997 82 2386–2390. (10.1210/jcem.82.8.4163) 9253305

[28] Hackett G Kirby M Edwards D Jones TH Wylie K Ossei-Gerning N David J & Muneer A. British society for sexual medicine guidelines on adult testosterone deficiency, with statements for UK practice. Journal of Sexual Medicine 2017 14 1504–1523. (10.1016/j.jsxm.2017.10.067) 29198507

[29] Wary C Azzabou N Giraudeau C Louër Le J Montus M Voit T Servais L & Carlier P. Quantitative NMRI and NMRS identify augmented disease progression after loss of ambulation in forearms of boys with Duchenne muscular dystrophy. NMR in Biomedicine 2015 28 1150–1162. (10.1002/nbm.3352) 26215733

[30] Hogrel JY Wary C Moraux A Azzabou N Decostre V Ollivier G Canal A Lilien C Ledoux I Annoussamy M, ***et al***. Longitudinal functional and NMR assessment of upper limbs in Duchenne muscular dystrophy. Neurology 2016 86 1022–1030. (10.1212/WNL.0000000000002464) 26888987 PMC4799716

